# The Book World of Medicine and Science

**Published:** 1906-09-29

**Authors:** 


### The Book World of Medicine and Science.

Guide to Anæsthetics for the Student and Practitioner. By Thomas D. Luke, M.B., F.R.C.S. (Ed.). Lecturer on Anæsthetics Aberdeen University. With 43 illustrations. Third edition. (William Green and Sons, London and Edinburgh.)

The third edition of any work is a good guarantee of its practical value for students and practitioners. The present volume contains but little fresh matter and very few emendations, except on the choice of an anæsthetic and the interesting part dealing with the practice of local anæsthesia. The volume is printed on art paper, and consequently the illustrations are well brought out. They consist of the various positions used in general anæsthesia, the ordinary nitrous oxide and other apparatus and cylinders, together with mouth-props and gags. The book is eminently practical, the language terse and pointed, and the general ensemble is an agreeable work from which some useful information can be got by all who are interested in the administration of anæsthetics. From the Lecturer at the Edinburgh School, where Simpson reduced the search for an agent for the abolition of pain into something approaching scientific order, very much is expected on this subject. When he has to deal with the administration of anæsthetics in the town of Edinburgh, which has formed ever since anæsthesia was known the centre of the world's supply of anæsthetics, Dr. Luke's task and responsibilities are greatly enhanced. That he has fulfilled them all in a very distinguished manner will be manifest by the speedy sale which this third edition is certain to attain. The section on ethyl-chloride is well set forth, and a description of the apparatus used is given, with a short account of its mode of administration. In an appendix he notices that owing to something like a scare which has been worked up over the administration of ethyl-chloride, some anæsthetists do not administer the drug. He is perfectly justified in saying that while it is impossible to understand such an attitude on the part of trained anæsthetists, there is no doubt that in unskilled hands and by a casual administrator there is much risk, and ethyl-chloride should be used with a great deal of caution both as regards dosage and administration.

The Prevention of Senility and a Sanitary Outlook. By Sir James Crichton-Browne, M.D., LL.D., F.R.S., Lord Chancellor's Visitor in Lunacy. (Macmillan and Co., Ltd., London.)

The two addresses which Sir James Crichton-Browne has delivered are so well known that it is scarcely necessary to do more than allude to the perennial interest which the diction and erudition of these lectures must always sustain. The lecture on the prevention of senility was called forth by the public excitement caused by a remark of Professor Osler that man's work was done at 40 years of age, and the aged should retire to a college at 60 for a year of contemplation before peaceful departure by chloroform. Sir James speedily shattered that position; indicted it as flying in the face of the biographical dictionary by showing that nearly all the great work of scientists, poets, and discoverers was accomplished after that age. No less interesting is the "Sanitary Outlook," in which Sir James deals with the general movement of public health, and he points out, in order to accomplish amelioration of the conditions of the people, strenuous efforts are needed. We can only hope that what he says to the sanitary inspectors will be merited by them by increased efforts in the discharge of their duties. Of them he says: "You have in a multiplicity of ways promoted that cleanliness which is not inferior to godliness in giving a man length of days in the land. You have sweetened our lives by curbing the cupidity of tradesmen and manufacturers. You have protected us from secret poisoning in our food on a scale that the Borgia never dreamed of. You have, at no small risk to yourselves, warded off from us contagious, infectious, and epidemic diseases, and extinguished sparks of them which but for you might have become ruinous conflagrations." If the sanitary inspectors do not always come up to this high level, or have the aspirations which Sir James sets before them, they can at least use his words as a guide, a stimulus, and a motive for more faithful fulfilment of duties entrusted to them.

